# Characterisation of Milk Microbiota from Subclinical Mastitis and Apparently Healthy Dairy Cattle in Free State Province, South Africa

**DOI:** 10.3390/vetsci10100616

**Published:** 2023-10-11

**Authors:** N. G. Khasapane, Z. T. H. Khumalo, S. Kwenda, S. J. Nkhebenyane, O. Thekisoe

**Affiliations:** 1 Centre for Applied Food Safety and Biotechnology, Department of Life Sciences, Central University of Technology, 1 Park Road, Bloemfontein 9300, South Africa; snkheben@cut.ac.za; 2 ClinVet International, Study Management, Bainsvlei, Bloemfontein 9300, South Africa; zamantungwa.khumalo@clinvet.com; 3 Vectors and Vector-Borne Diseases Research Programme, Department of Veterinary Tropical Diseases, Faculty of Veterinary Science, University of Pretoria, Onderstepoort, Pretoria 0110, South Africa; 4 Sequencing Core Facility, National Institute for Communicable Diseases, National Health Laboratory Service, Johannesburg 2192, South Africa; stanfordk@nicd.ac.za; 5 Unit for Environmental Sciences and Management, North-West University, Potchefstroom 2531, South Africa; oriel.thekisoe@nwu.ac.za

**Keywords:** subclinical mastitis, microbiota, metabarcoding, cattle milk

## Abstract

**Simple Summary:**

Mastitis is an inflammation of the mammary gland caused by mechanical, physical, chemical, and biological causes. The identification of mastitis causative agents was accomplished using microbiological culture and DNA techniques, a common method in veterinary medicine. Moreover, significant advancements in high throughput next generation sequencing (NGS) and bioinformatics techniques have facilitated the transition from culture-based methods to genomic sequence-based characterisation of the microbiome associated with mastitis infection in both humans and animals. Hence, this study utilised 16S metagenomics to understand the taxonomic profiles of both subclinical mastitis (SCM) and apparently healthy (non-SCM) dairy cow milk of small-scale farmers. Overall, the analysis indicated a total of 95 phyla, 33 classes, 884 orders, 124 families, 202 genera, and 119 bacterial species. The data analysis clearly indicates that the microbiome composition in SCM and non-SCM cows is considerably different.

**Abstract:**

Bovine mastitis is an inflammation of the udder tissue of the mammary gland brought on by microbial infections or physical damage. It is characterised by physical, chemical, and biological changes in the udder and milk. While several different bacterial species have been identified as causative agents of mastitis, many subclinical mastitis (SCM) cases remain culture-negative. The aim of this study was to characterise milk microbiota from SCM and apparently healthy dairy cows (non-SCM) by 16S rRNA sequencing. Alpha-diversity metrics showed significant differences between SCM cows and non-SCM counterparts. The beta-diversity metrics in the principal coordinate analysis significantly clustered samples by type (PERMANOVA test, *p* < 0.05), while non-metric dimensional scaling did not (PERMANOVA test, *p* = 0.07). The overall analysis indicated a total of 95 phyla, 33 classes, 82 orders, 124 families, 202 genera, and 119 bacterial species. Four phyla, namely Actinobacteriota, Bacteroidota, Firmicutes, and Proteobacteria collectively accounted for more than 97% of all sequencing reads from SCM and non-SCM cow samples. The most abundant bacterial classes were Actinobacteria, Bacilli, Bacteroidia, Clostridia, and Gammaproteobacteria in non-SCM cow samples, whilst SCM cow samples were mainly composed of Actinobacteria, Alphaproteobacteria, Bacilli, Clostridia, and Gammaproteobacteria. Dominant bacterial species in non-SCM cow samples were *Anthropi* spp., *Pseudomonas azotoformans*, *P. fragi*, *Acinetobacter guillouiae*, *Enterococcus italicus*, *Lactococcus lactis*, whilst *P. azotoformans*, *Mycobacterium bovis*, *P. fragi*, *Acinetobacter guillouiae*, and *P. koreensis* were dominant in the SCM cow samples. The current study found differences in bacterial species between SCM and non-SCM cow milk; hence, the need for detailed epidemiological studies.

## 1. Introduction

Mastitis is regarded as a significant illness with a global impact on the dairy business, including medicine and disease management expenses, death among sick animals, and recurrence costs [1]. The most common kind of mastitis, subclinical mastitis, is an asymptomatic form of intramammary inflammation that affects 20–50% of cows in a particular herd. Cows are considered infected when they have at least 2 out of 3 weekly composite SCC results >150 × 10^3^ cells/mL. Subclinical mastitis causes little changes in milk characteristics, although it may include germs that cause the illness. Subclinical mastitis causes more losses than clinical mastitis and is more likely to spread across individual cows in a given herd depending on the mastitis-causing pathogen [2,3]. The sudden onset of this infection is mainly due to the entry of infective bacteria into the udder, leading to the rupture of the glands’ physical barriers, necessitating immediate and effective host defences to avert colonisation and further disease pathology [2]. When the illness advances, dysbiosis of the milk microbiome occurs, in which milk has an increasing number of pathogenic bacteria and fewer commensal bacteria. Until recently, description of microbiota associated with mastitis relied on the discovery and isolation of a single bacterium [3].

Due to the fact that the condition is caused by epidemiologically distinct microorganisms, it can be characterised as contagious or environmental mastitis depending on the causal agents [4]. *Staphylococcus aureus*, *Streptococcus agalactiae*, *Streptococcus dysgalactiae*, *Mycoplasma* spp., and *Corynebacterium bovis* are among the organisms that cause contagious mastitis [5], but *Escherichia coli* is the most common cause of environmental mastitis [6]. Despite the extensive understanding of bacteria that cause mastitis in cows, Lin et al. [7] emphasized that additional microorganisms such as archaea, viruses, and fungi may be involved in the disease process and should be explored as well [8]. 

One of the most important aspects of the fight against the disease is being able to swiftly, accurately, and precisely detect mastitis [9]. The traditional approaches typically disregard the element of mastitis causation, concentrating instead on symptomatic diagnosis. A lack of focused therapy, improper antibiotic selection, and overuse of antibiotics all contribute to the development of drug resistance in bacteria and raise the risk of antibiotic contamination of milk [10,11]. The identification of these causative agents has been accomplished using microbiological culture, a common methodology in veterinary medicine [10]. Even though the microbiological culture of causative agents does not always result in bacterial growth, molecular approaches can enhance the detection of mastitis with high sensitivity and specificity [12]. Significant advancements in high throughput next generation sequencing (NGS) and bioinformatics techniques have facilitated the transition from culture-based methods to genomic sequence-based characterisation of the microbiome associated with mastitis infection in both humans and animals [13,14]. Amongst these NGS approaches, 16S rRNA gene sequencing has remained the most extensively utilised technique in characterising mastitis microbiota in recent years [15]. These studies also provided insight into the functional characteristics of these microbial communities, including information on microbial metabolism, pathogenicity, and antibiotic resistance [16,17]. A review of studies on milk microflora clearly illustrates the common taxa present in cow’s milk from many locations. Among the most often stated dominating taxa in research on the microbiota of bovine milk are *Staphylococcus*, *Streptococcus*, *Pseudomonas*, *Bifidobacterium*, *Propionibacterium*, *Bacteroides*, *Corynebacterium*, and *Enterococcus* [18,19,20]. Furthermore, metataxonomic investigations have revealed alterations in the population of pathogenic bacteria that cause mastitis [21], lactic acid bacteria (LAB) [22], and spoiled milk bacteria [23]. Hence, the current study used 16S rRNA metabarcoding to characterise and compare milk microbiota of subclinical mastitis (SCM) and apparently healthy (non-SCM) dairy cows of small-scale farms in the Free State province of South Africa.

## 2. Materials and Methods

### 2.1. Mastitis Screening and Sample Collection

Samples were collected according to national mastitis council guidelines [24]. Milk samples were collected at the same time of day (early in the morning) following the customary pre-milking udder preparation by the farmer or farm workers and before attachment of the milking machine. Subsequently, before sample collection, the individual teats of each cow were washed with distilled water and dried with disposable paper towels to prevent any cross-contamination. Thereafter, to ensure that samples were collected aseptically, 70% ethanol was applied on teat ends before milk samples were collected using 50 mL sterile falcon tubes. The initial milk streaks were discarded. 

**Figure 1:**
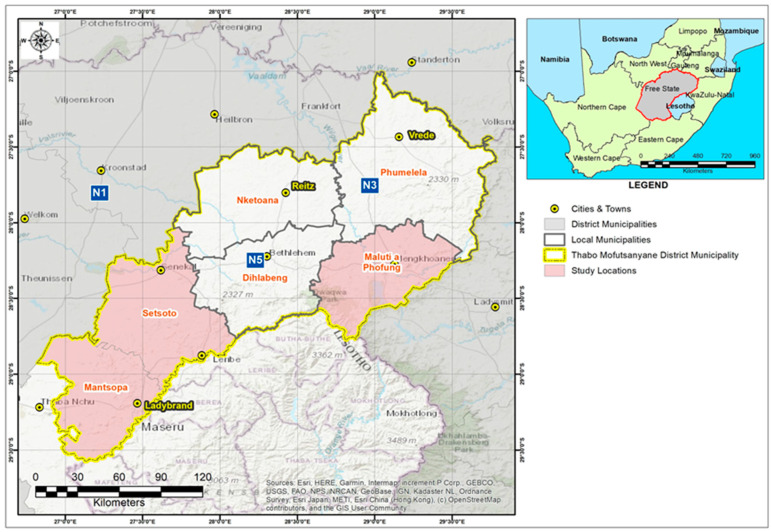
Map showing Free State province (yellow line) within South Africa and the sampled municipalities (pink colour) within Free State province.

A total of 166 composite milk samples from 166 individual cows of seven small-scale farms located in three local municipalities, namely Maluti-a-Phofung, Mantsopa, and Setsoto (Figure 1), were randomly screened and collected for intramammary infection examination by means of California mastitis test according to manufacturer’s instructions (DeLaval, South Africa). Briefly, after foremilk was discarded, one or two squirts of composite milk were collected in each paddle compartment per cow. The paddle was slanted to enable the majority of the milk to drain, leaving 1 to 2 teaspoons (5 to 10 mL) in each compartment. A volume of CMT reagent equal to the withheld milk was introduced to each cell. The milk reagent combination was whirled in a circular motion, and the presence of gel or slime was visually observed and recorded for each quarter. In summary, scores were assigned on a scale of 0 to 4, with 0 representing no reaction, 1 representing a trace, 2 representing a weak positive, 3 representing a definite positive, and 4 representing a strong positive. Thereafter, positive samples were then subjected to somatic cell count (SCC) assay using flow cytometry (Mérieux NutriSciences, Midrand, South Africa). Thereafter, based on the SCC results, only 10 samples from 55 cows were diagnosed with subclinical mastitis and 10 samples from 166 samples that were considered healthy were collected using sterile 50 mL falcon tubes and transported to the laboratory for DNA extraction using cooler box maintained with ice. Somatic cell counts results were scored and interpreted as weak positive, SCC > 100,000–500,000 cells/mL milk, distinct positive, SCC > 500,000–1,000,000 cells/mL milk, and strong positive, SCC > 1,000,000 cells/mL milk, as recommended by Karzis et al. [24]. Hence, samples that had SCC of >100,000 < 500,000 cells/mL and CMT 1+ were considered to be subclinically infected while those that had SCC of ≤100,000 cells/mL milk and CMT 0 were considered to be healthy.

### 2.2. DNA Extraction and PCR

For the purpose of this study, Quick-DNA™ Fungal/Bacterial Miniprep Kit was used to extract genomic DNA from respective milk samples according to manufacturer’s instructions (Zymo Research, Irvine, CA, USA). A NanoDrop™ 2000 Spectrophotometer was used to measure the DNA concentration (Oxoid, Thermo Fisher, Johannesburg, South Africa).

### 2.3. 16S rRNA Gene Amplification and Sample Barcoding

Utilising single-molecule real-time PacBio sequencing technology (Pacific Biosciences, Menlo Park, CA, USA), the diversity of bacterial populations in milk samples from diverse farms was examined. The bacterial-specific primers 27F (5′-AGAGTTTGATCMTGGCTCAG-3′) and 1492R (5′-TACGGYTACCTTGTTACGACTT-3′) were used to amplify the full-length 16S ribosomal RNA gene from genomic DNA, as reported by Pootakham et al. [25] at Inqaba Biotechnical Industries (Pty) Ltd., Pretoria, South Africa. In addition to processing the experimental samples, one reference strain, or UHT milk, was also treated. It included the same PCR and sequencing laboratory equipment as the experimental samples, but the experimental DNA template was replaced with double-distilled water. An absence of contamination by chemicals that may affect the results was shown by the failure of a negative control to amplify in each PCR reaction during sequencing, as seen in the work of Catozzi et al. [16].

### 2.4. Bioinformatics and Statistical Analyses

The DADA2 analysis workflow [26], implemented in R software (v 4.3.1) package [27], was used to analyse raw amplicon sequencing data generated using the PacBio Sequel System (Pacific Biosciences, Menlo Park, CA, USA). Briefly, raw amplicon data were initially processed using the PacBio SMRT Link software (v 7.0) to generate consensus circular sequence (CCS) reads in FASTQ format. As part of the DADA2 workflow, primer sequences were filtered, and the PacBio CCS reads subjected to quality control following the standard DADA2 pre-processing steps for PacBio CCS reads (https://www.biorxiv.org/content/10.1101/392332v2.full, accessed on 17 January 2022). Inference of the amplicon sequence variants (ASVs) was performed using the DADA2 method. Taxonomy and ASV abundances were determined and assigned using DADA2-formatted training sequence sets based on the SILVA reference database (v138; https://zenodo.org/record/4587955#.Y2F0p3ZBw2w accessed on 17 January 2022).

All downstream analyses, including abundance bar plots, alpha and beta diversity ordinations, richness and differential abundance estimates, statistical analysis, and visualisations, were performed using Phyloseq (v1.28.0) [28], ggplot2 (v3.2.1), AmpVis2 (v2.6.4) [29], and DESeq2 (v1.24.0) [22] packages in R (v3.6.1). Group-level significant differences in NMDS ordination plots were assessed using PERMANOVA (permutation test with pseudo-F ratios) as implemented in the adonis function in the vegan package (https://github.com/vegandevs/vegan accessed on 17 January 2022). To compare alpha diversity between groups, Kruskal–Wallis rank-sum tests were utilised. UpsetR (v1.4.0) and Venn Diagram (v1.6.20) were used to generate Venn diagrams [30].

## 3. Results

### 3.1. Somatic Cell Counts

Our findings revealed that, out of 166 cows evaluated for subclinical mastitis, only 55 (33.13%) had SCC > 200,000 < 500,000 cells/mL milk and were thought to suffer from inflammation of the mammary gland at a cow level, based on the criterion established by the National Mastitis Council’s recommendations and Karzis et al. [24].

### 3.2. Characteristics of the Sequences

An overall of 499,544 (minimum = 9152; mean = 24,977.2; maximum = 48,172) CCSs of 10 raw milk samples from cows that were not subclinically inflamed and 10 samples from cows that had subclinical mastitis were used to produce a total of 209,357 (minimum = 3869; mean = 10,467.85; maximum = 25,205) non-chimeric Illumina reads. Through DADA2, the readings from the analysed sample groups were combined into 883 unique ASVs at the Kingdom (Bacteria) level. A recognised phylum could be ascribed to at least 98.8% of the sequences (Table S1). 

### 3.3. Taxonomic Profile

**Figure 2:**
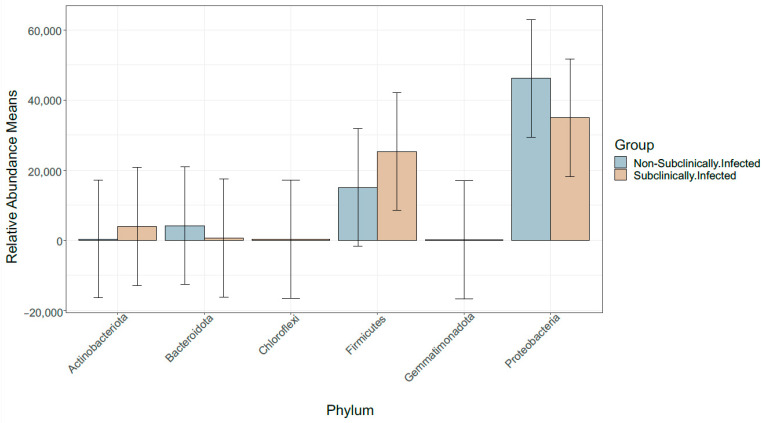
Distribution of four most abundant phyla in SCM and non-SCM milk samples.

To obtain precise bacterial profiles of raw milk associated with subclinical mastitis in comparison to bacterial profiles of non-subclinical mastitis raw milk, SMRT sequencing of the full-length 16S rRNA gene was conducted. The ASVs were observed from 17 bacterial phyla with an average relative abundance of 5.8% and four of these phyla (Actinobacteriota, Bacteroidota, Firmicutes, and Proteobacteria) collectively accounted for more than 97% of all sequencing reads of infected and healthy samples (Figure 2).

**Figure 3:**
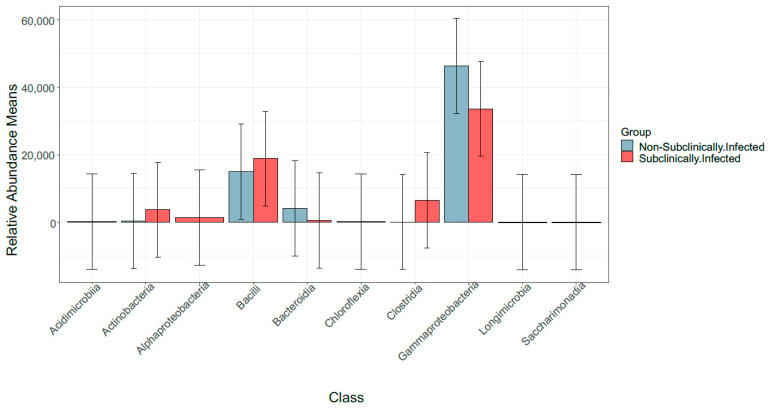
Distribution of five most abundant classes in SCM and non-SCM cow milk samples.

Further analysis of the core microbiome identified most abundant bacterial classes as Actinobacteria, Bacilli, Bacteroidia, Clostridia, and Gammaproteobacteria in non-SCM samples and in SCM samples as Actinobacteria, Alphaproteobacteria, Bacilli, Clostridia, and Gammaproteobacteria (Figure 3). It is worth noting that our reference sample showed a rather different taxonomic profiling compared to non-SCM and SCM samples. Our results show that Kingdom classification indeed corresponded to Bacteria, phylum Bacteroidota, class Bacteroidia, bacterial order Flavobacteriales, family *Weeksellaceae*, genus *Chryseobacterium,* and one species which were identified, i.e., *Chryseobacterium hominis* (Table S2).

**Figure 4:**
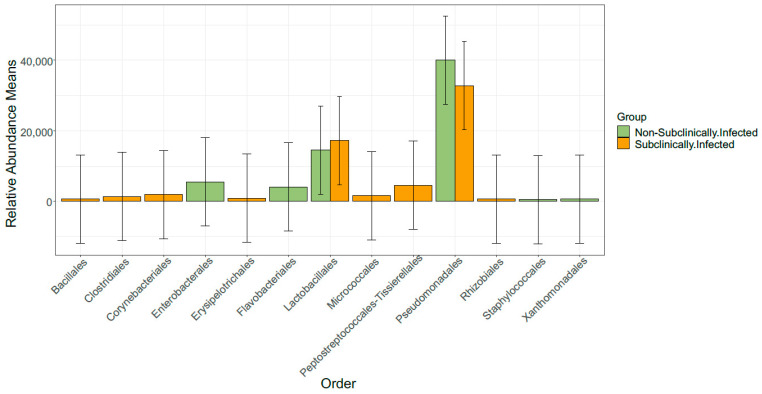
Distribution of five most abundant bacterial orders in SCM and non-SCM cow milk samples.

**Figure 5:**
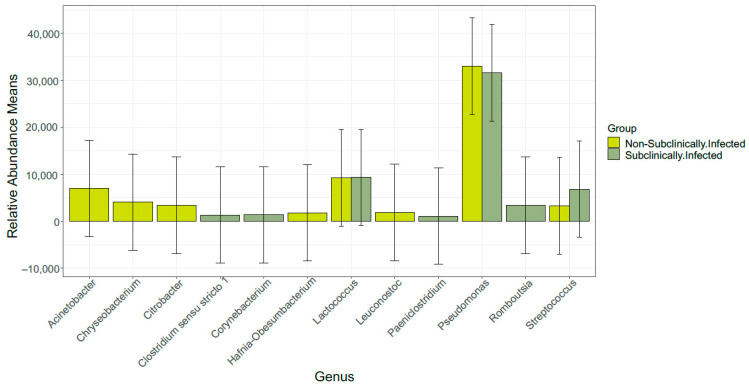
Distribution of fifteen most abundant genera in SCM and non-SCM cow milk samples.

This study further observed that the order of the bacteria was diverse with the non-SCM samples containing an order of Enterobacterales, Flavobacteriales, Lactobacillales, Pseudomonadales, and Xanthomonadales, while the SCM samples, on the other hand, contained Corynebacteriales, Erysipelotrichales, Lactobacillales, Peptostreptococcales–Tissierellales, and Pseudomonadales, respectively (Figure 4). Subsequently, we observed that both groups of samples were composed of 15 bacterial genera, respectively, which, amongst others, included the following: *Acinetobacter*, *Pseudomonas*, *Clostridium sensu stricto 1 Corynebacterium*, *Dietzia*, *Enterococcus*, *Kocuria*, *Lactococcus*, *Leuconostoc*, *Methylobacterium–Methylorubrum*, *Paeniclostridium*, *Romboutsia*, *Sphingomonas*, *Streptococcus*, and *Turicibacter* (Figure 5).

**Figure 6:**
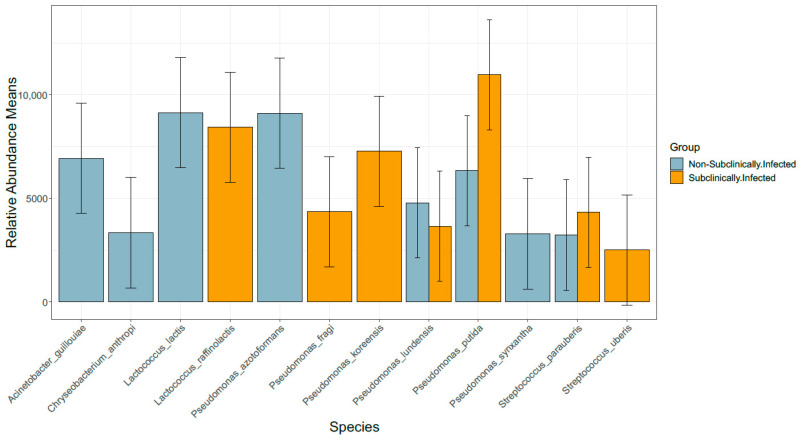
Distribution of fifteen most abundant species in SCM and non-SCM cow milk samples.

Lastly, the sequences were analysed at the species level, and we found that both groups contained 15 dominant bacterial species that were diverse. The bacterial species *Anthropi*, *P. azotoformans*, *P. fragi*, *A. guillouiae*, *E. italicus*, *L. lactis*, *P. lundensis*, *L. mesenteroides*, *C. otitidis*, *S. parauberis*, *psychrophile*, *P. putida*, *rhizophila*, *C. shigense*, and *P. synxantha* were found in non-SCM samples while, on the other hand, *P. azotoformans*, *M. bovis*, *P. fragi*, *A. guillouiae*, *P. koreensis*, *L. lactis*, *P. lundensis*, *M. marinum*, *L. mesenteroides*, *S. parauberis*, *P. putida*, *L. raffinolactis*, and *P. synxantha* were found in SCM samples (Figure 6).

### 3.4. Alpha Diversity Determined by 16S Sequencing

When comparing the alpha diversity of the complete groups of samples, the species richness indices (Chao1 and ACE) and species evenness (Shannon and Simpson) indices indicated that SCM and non-SCM cow milk samples were not significantly different between the groups. Both Chao1 and ACE indices did not indicate a significant difference between SCM and non-subclinical mastitis samples (Supplementary Figures S1 and S2). On the other hand, the Shannon diversity indices of the groups (SCM and non-subclinical mastitis) did not differ (Kruskal–Wallis One-way ANOVA: *p* = 0.58) (Supplementary Figure S3) and the Simpson’s diversity was not significantly different either (Kruskal–Wallis One-way ANOVA: *p* = 0.8) (Supplementary Figure S4). Rarefaction results confirm the variety in the data, with some of the healthy samples having the highest diversity of all samples.

### 3.5. Beta Diversity Analysis Determined by 16S Sequencing

For the purpose of this study, two β-diversity analyses were performed, and both had the same patterns. NMDS analysis showed that bacterial communities from SCM and non-SCM cow milk samples were not clustered together; hence, they were not significantly different to each other (PERMANOVA *p* = 0.07; F = 1.53) (Supplementary Figure S5). Principle co-ordinates analysis confirmed the NMDS analysis indicating the (PERMANOVA) *p* = 0.05; F = 1.53 (Supplementary Figure S6).

### 3.6. Comparison of Bacterial Taxonomy Overlaps and Differential Abundance in SCM and Non-SCM 

**Figure 7:**
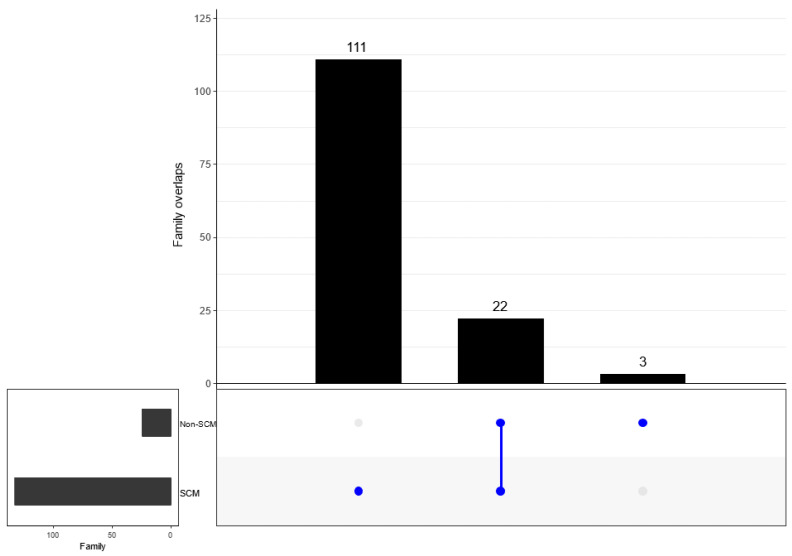
UpSetR intersection plot showing number of unique and shared taxa at family level between non-SCM and SCM groups. One dot indicates number of unique taxa, while two dots indicate number of shared taxa at a family level.

**Figure 8:**
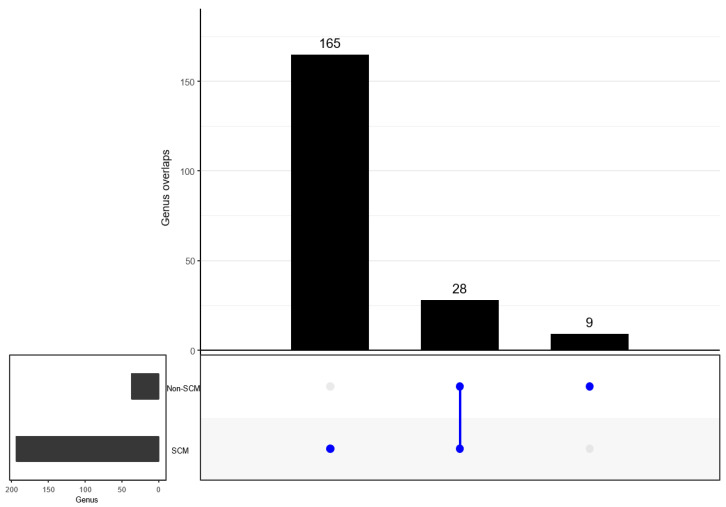
UpSetR intersection plot showing number of unique and shared taxa at genus level between non-SCM and SCM as “infected” groups. One dot indicates number of unique taxa, while two dots indicate number of shared taxa at a genus level.

**Figure 9:**
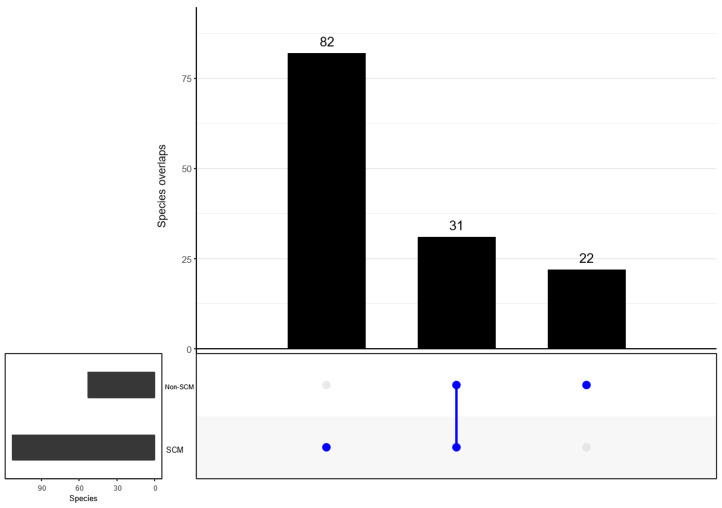
UpSetR intersection plot showing number of unique and shared taxa species level between non-SCM and SCM as “infected” groups. One dot indicates number of unique taxa, while two dots indicate number of shared taxa at a species level.

When determining the overlap in the detected bacterial taxonomy, both groups (SCM and non-SCM) of cow milk samples shared 22 families. However, 111 and 3 families were uniquely detected in SCM and non-SCM cow milk samples (Figure 7). When examining overlapping genera, both groups shared 28 genera. Separately, the SCM sample group had 165 genera alone, while the non-SCM sample group had nine non-shared genera (Figure 8). When examining species composition, the SCM samples had 82 unique species while the non-SCM samples had 22 and they both shared 31 species (Figure 9). When examining differential abundance between SCM and non-SCM samples, all had Firmicutes, Actinobacteriota, and Proteobacteria as dominant phyla. However, Romboutsia, Corynebacterium, Pseudomonas, and Clostridium sensu stricto 1 were most abundant genera and Acinetobacter was the least abundant.

## 4. Discussion

Mastitis research in general and the quest for the causes of its development remain important to date, and the application of omics technology to find answers to these problems has been very limited [31]. Subclinical mastitis is a disease that frequently attacks lactating dairy cows, causing economic losses to farmers due to decreased milk production [32]. The occurrence of SCM in nursing cows has also been linked to the onset and progression of clinical mastitis. Furthermore, knowing the causes of mastitis and the microorganisms that cause it would aid in the development of preventative strategies to lower its incidence. The current study used an SMRT sequencing approach to compare milk microbiota associated with bovine subclinical mastitis from healthy cows. Various research studies have been undertaken utilising various sequencing technologies to comprehend the bacterial communities; nevertheless, they are restricted to just identifying microorganisms at the genus level [33,34,35]. Sequencing technologies, such as PacBio SMRT sequencing, provide advantages over other sequencing platforms, such as the ability to generate longer reads or full-length sequences and resolution that allow for the identification of bacteria at the species level; hence, it was utilised in this investigation. 

Since 1960, somatic cell counts (SCCs) from composite milk samples have been widely accepted and used as the operational measure of inflammation of the bovine lactating gland [36] and as an indicator of economic losses [37]. The SCC threshold level was used as indicator of udder health, which has been and continues to be a contentious issue [17]. As a result, from a South African perspective, this study considered the Karzis et al. [24] criterion, which considers cows to be subclinically inflamed if they have an SCC of >100,000 < 500,000 cells/mL milk. The bovine milk DNA was utilised to better understand the microbiome of dairy cows suspected of having subclinical mastitis in comparison to healthy cows (non-SCM). Proteobacteria, Firmicutes, Actinobacteria, and Bacteroidetes dominated the bovine milk microbiome across both groups, and their relative abundance changed among samples of SCM and non-SCM dairy cow milk samples. These findings are consistent with previous studies in bovine [38,39] and human [40] mastitis microbiomes. Proteobacteria and Firmicutes were previously identified as the predominant phyla in bovine SCM [41], which supports our recent findings.

In this study, the SCM samples were dominated by several strains of *Pseudomonas*, *Lactococcus*, and *Streptococcus*, supporting the findings of previous studies conducted in Poland from raw cow’s milk samples [42]. Many of these microbiomes may operate as potential opportunists, interfering with metabolism, host defence, and immunological development [43], resulting in udder infections of varying severity [44]. Our research also revealed the existence of Methylobacterium, a genus of strictly aerobic, Gram-negative bacteria found in soil, freshwater, and lake sediments [45]. These bacteria have been linked to opportunistic infections in immunocompromised individuals [46]. Methylobacterium was also found in milk samples [47]; however, no findings have linked it to bovine mastitis. *Streptococcus* and *Corynebacterium* spp. were found to have higher relative abundances among the dominating genera in both groups. *Streptococcus* spp., such as *S. agalactiae*, *S. uberis*, and *S. dysgalactiae*, are well-known bacteria that cause mastitis [48,49].

*S. parauberis*, previously known as *Streptococcus uberis* type II [50], was found in some of the samples. It is most known for causing mastitis in cows, an inflammatory illness that affects the bovine mammary glands, and is isolated in up to 20% of cases. *Corynebacterium* spp. was also identified as the bacteria responsible for mastitis in dairy cows, and it is frequently described as contagious [51]. Previous research found Corynebacterium in bulk tank milk samples obtained from 894 China dairy herds at a 17.0% frequency [52] and from 1242 dairy cows in Brazil at a 22.9% frequency [53]. Microbiome diversity (alpha and beta diversity) measurements revealed that microbial dysbiosis differed between healthy and diseased samples. The current study revealed differences in microbial diversity and species richness from SCM and non-SCM cow milk samples. Beta diversity [54] also demonstrated a significant microbial difference between SCM and non-SCM cow milk samples.

The dominant bacterial species found in SCM cow samples was *Pseudomonas*. This genus contains Gram-negative bacteria that is abundantly widespread in nature and is thought to be the most frequent dairy product spoiler [55]. Although *Pseudomonas* spp. are not regarded as important human or animal pathogens, numerous species of this genus have been linked to human and animal diseases. Due to a lack of suitable identification methods for these organisms, non-pathogenic *Pseudomonas* spp. (*P. psychrophila*, *P. putida*, *P. koreensis*, among others) have been misidentified as pathogenic [56].

Several *Lactococcus* spp. (*Lactococcus raffinolactis* and *Lactococcus lactis*) were also found in this study. *Lactococcus* bacteria are considered to infect humans via the gastrointestinal system [57]. The pathogenicity of *L. lactis* subsp. *cremoris*, as well as the method of infection, remain unknown [58]. Other studies that used culture-independent methodologies found *Lactococcus* in raw milk, pasteurised milk, and raw milk from bovine mastitis [59,60]. As a consequence, the prevalence of the *Lactococcus* genus in SCM and non-SCM samples was examined in this study. Lactococcus spp. were shown to be more abundant in mastitic milk, indicating that these organisms may be disease causative factors.

This current study also discovered *Leuconostoc mesenteroides*. This bacteriocin inhibited additional *Leuconostoc* strains as well as various *Enterococcus* and *Listeria* spp. strains. The majority of lactic acid bacteria (LAB) are found in qualified presumption of safety (QPS) and generally recognised as safe (GRAS) lists, ensuring their food safety [61]. However, some LAB, such as Enterococcus sp., are excluded from these benefits due to their roles in causing certain human infections and contributing to the spread of antibiotic resistance [62], and their presence in milk could indicate unsanitary production and faecal pollution of either human or animal routes, or both, because they are ubiquitously found in the intestinal microflora of humans and animals [63].

This study also found *Acinetobacter* spp. (*A. guillouiae*), which is of importance because little is known about the role of foods in the transmission of *Acinetobacter* spp., most likely because there are no standard protocols for recovering them from foods [64,65]. Many articles, however, documented the presence of *Acinetobacter* spp. in raw and pasteurised bovine milk, dairy products, and powdered milk [66,67]. *Acinetobacter* spp. are common microbes found throughout nature. As a result, their presence in raw milk may simply be attributable to contamination. *Acinetobacter* spp. can contaminate bulk tank milk from teats, udder surfaces, diseased mammary glands, milking machines, transportation systems, and contaminated water used to clean the milking equipment, as well as from the dairy farm environment [68]. Finally, diverse strains of ambient, gastrointestinal, and animal skin-originating microorganisms dominated the healthy-milk microbiome. Though the pathogenic processes of these commensal bacteria are mostly unknown, they can induce opportunistic infections in the mammary glands and/or quarters with or without differing degrees of clinical episodes by creating diverse virulence factors, particularly in immunocompromised hosts [69].

## 5. Conclusions

This study used 16S metabarcoding to characterise the milk microbiome of SCM dairy cows in comparison to milk from non-SCM dairy cows. The data analysis clearly indicated that the microbiome composition in SCM and non-SCM cows is considerably different. The current study found significant differences in specific dominating circular consensus sequencing (CCS) in milk samples that had never been reported in previous investigations. Alpha-diversity analysis showed that the richness and diversity in microbiota in SCM samples were greater than in non-SCM samples. Based on milk microbiota observed from SCM cow milk in this study, detailed epidemiological studies are recommended for bovine mammary gland health management, which can give supplemental information such as raw milk microbiota ecology and the identification of fastidious bacteria and polymicrobial illnesses.

## Data Availability

The data used to support the findings of this study are available in the present manuscript.

## Supplementary Materials

The following supporting information can be downloaded at: https://www.mdpi.com/article/10.3390/vetsci10100616/s1. Table S1: Read counts tracked through the DADA2 pipeline including ASV counts, richness, and genus-level resolved ASVs per sample. Table S2: Taxonomic profiling of reference sample. Figure S1: Alpha diversity boxplots showing Chao1 richness estimates (*p* = 0.47). Figure S2: Alpha diversity box = plots showing ACE indicators per group (SCM and Healthy cows) (*p* = 0.28). Figure S3: Alpha diversity boxplots showing Shannon’s diversity (*p* = 0.58). Figure S4: Alpha diversity boxplots showing Simpson’s diversity estimates (*p* = 0.8). Figure S5: Beta diversity of NMDS analysis of healthy and SCM groups (*p* = 0.07; F = 1.53). Figure S6: Shows principle co-ordinated analysis against SCM and heathy groups * (*p* = 0.05; F = 1.53).
