# Supplementary figures and images for: Characterisation of Milk Microbiota from Subclinical Mastitis and Apparently Healthy Dairy Cattle in Free State Province, South Africa
Source: Vet Sci. 2023 Oct 11;10(10):616. doi: 10.3390/vetsci10100616 (PMC10610705; doi:10.3390/vetsci10100616)

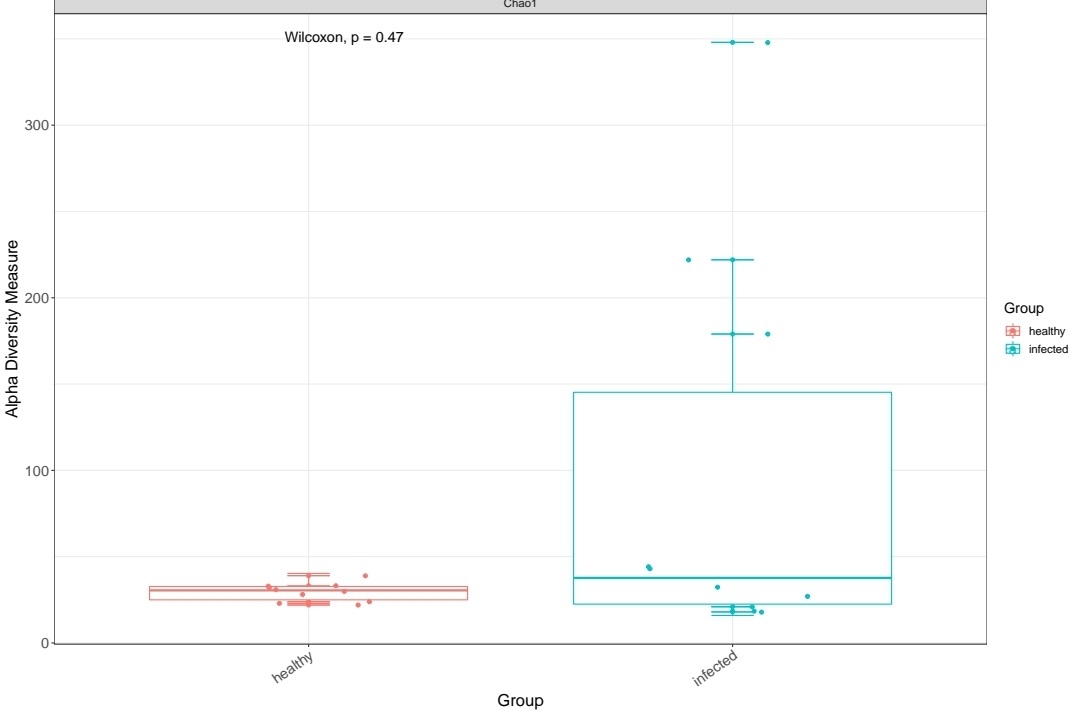

Supplement: Supplementary file 1 [file vetsci-10-00616-s001.zip › figure S1.jpg]

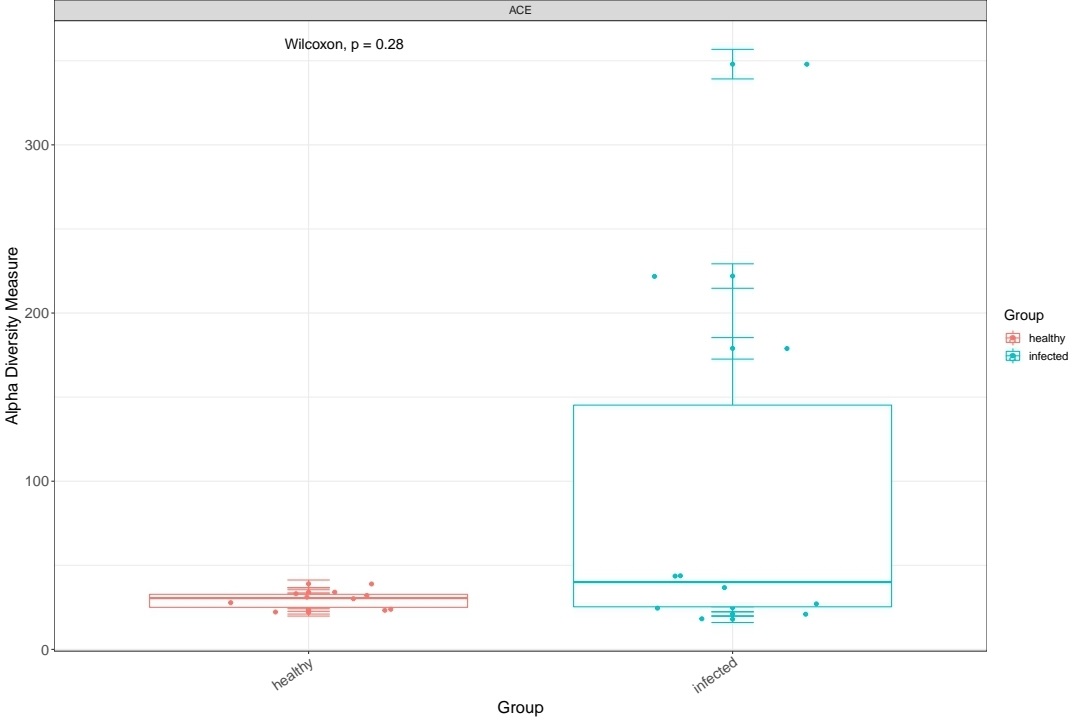

Supplement: Supplementary file 1 [file vetsci-10-00616-s001.zip › Figure S2.jpg]

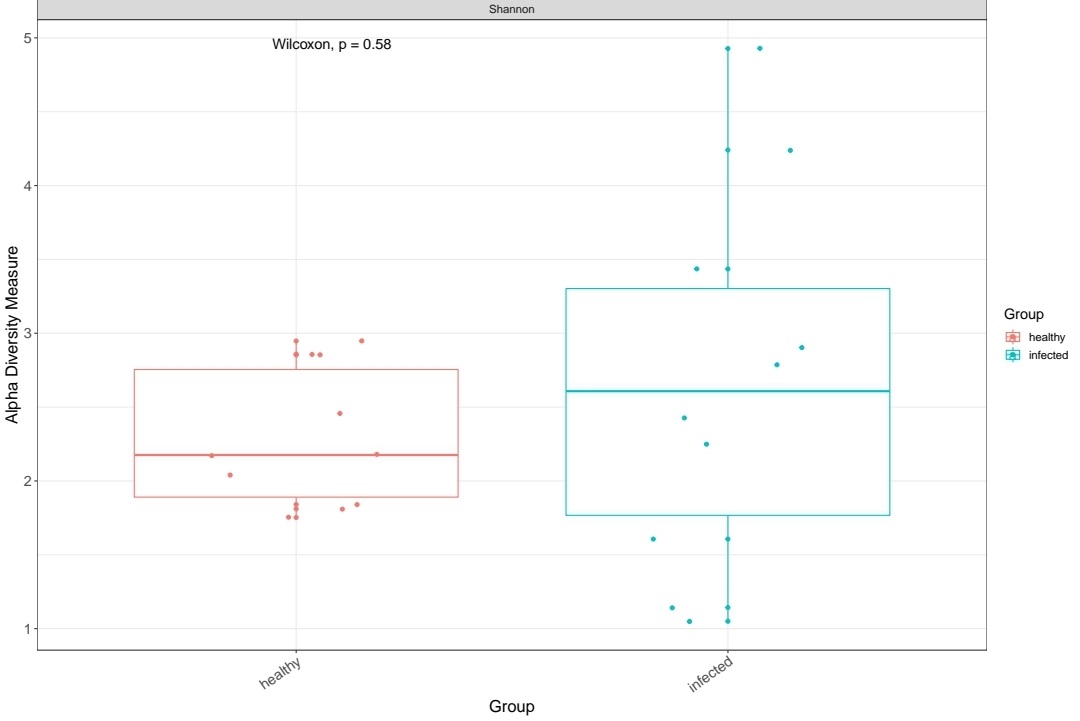

Supplement: Supplementary file 1 [file vetsci-10-00616-s001.zip › Figure S3.jpg]

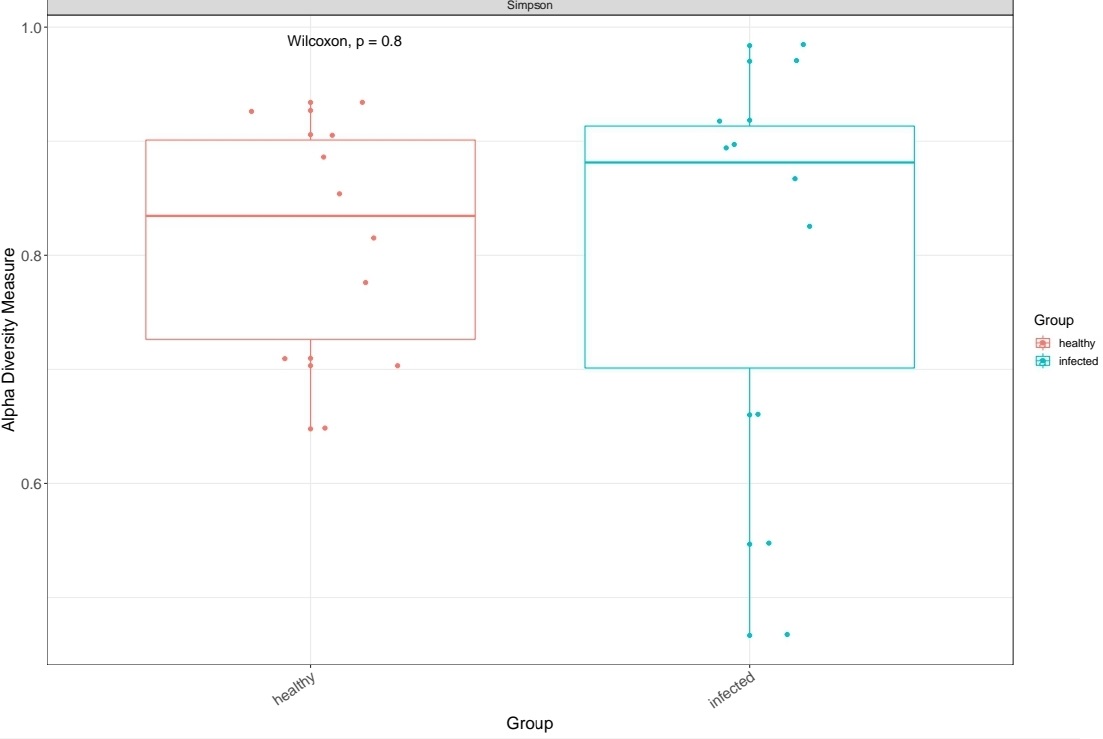

Supplement: Supplementary file 1 [file vetsci-10-00616-s001.zip › Figure S4.jpg]

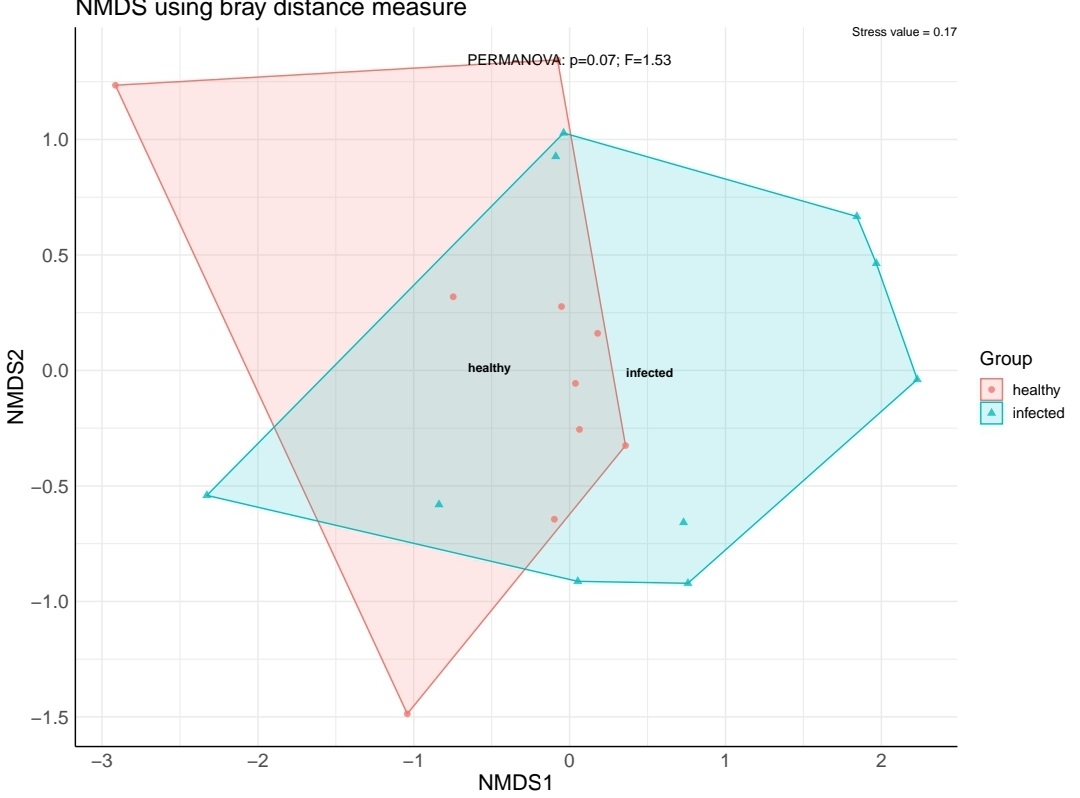

Supplement: Supplementary file 1 [file vetsci-10-00616-s001.zip › Figure S5.jpg]

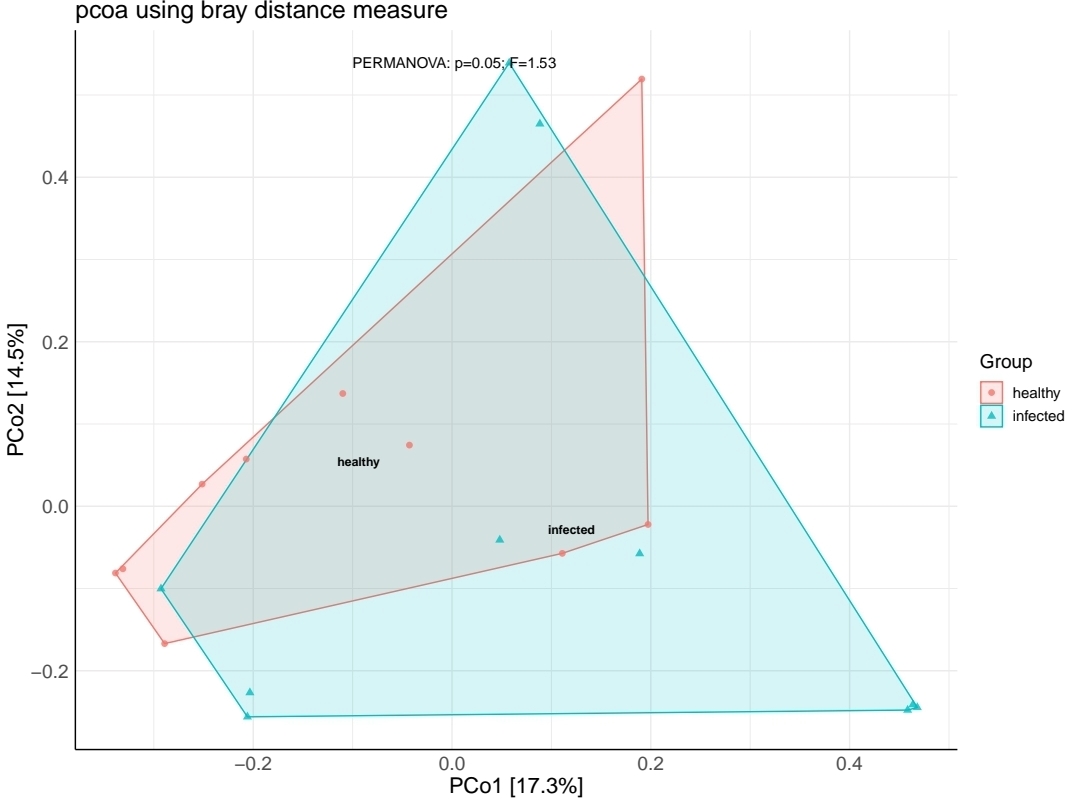

Supplement: Supplementary file 1 [file vetsci-10-00616-s001.zip › Figure S6.jpg]
